# Advances in research on fat infiltration and lumbar intervertebral disc degeneration

**DOI:** 10.3389/fendo.2022.1067373

**Published:** 2022-12-07

**Authors:** Zairan Wang, Zijun Zhao, Shiyuan Han, Xianghui Hu, Liguo Ye, Yongning Li, Jun Gao

**Affiliations:** ^1^ Department of Neurosurgery, Peking Union Medical College Hospital, Chinese Academy of Medical Sciences, Beijing, China; ^2^ Spine Center, Sanbo Brain Hospital, Capital Medical University, Beijing, China; ^3^ Department of International Medical Services, Peking Union Medical College Hospital, Chinese Academy of Medical Sciences, Beijing, China

**Keywords:** low back pain, intervertebral disc degeneration, paraspinal muscles, fatty infiltration, Modic changes, inflammation

## Abstract

Low back pain (LBP) is a disabling condition with no available cure, severely affecting patients’ quality of life. Intervertebral disc degeneration (IVDD) is the leading cause of chronic low back pain (CLBP). IVDD is a common and recurrent condition in spine surgery. Disc degeneration is closely associated with intervertebral disc inflammation. The intervertebral disc is an avascular tissue in the human body. Transitioning from hematopoietic bone marrow to bone marrow fat may initiate an inflammatory response as we age, resulting in bone marrow lesions in vertebrae. In addition, the development of LBP is closely associated with spinal stability imbalance. An excellent functional state of paraspinal muscles (PSMs) plays a vital role in maintaining spinal stability. Studies have shown that the diminished function of PSMs is mainly associated with increased fat content, but whether the fat content of PSMs is related to the degree of disc degeneration is still under study. Given the vital role of PSMs lesions in CLBP, it is crucial to elucidate the interaction between PSMs changes and CLBP. Therefore, this article reviews the advances in the relationship and the underlying mechanisms between IVDD and PSMs fatty infiltration in patients with CLBP.

## Introduction

Low back pain (LBP) is a disabling condition with no available cure, often caused by a sedentary lifestyle and reduced exercise (1), severely affecting patients’ quality of life. Chronic low back pain (CLBP) accounts for approximately 23% of LBP (2). Intervertebral disc degeneration (IVDD) is the leading cause of CLBP (3). IVDD is a chronic, multifactorial and irreversible process that severely compromises spinal stability and disc shock absorption (3). Early biochemical changes in IVDD include loss of proteoglycans and water, while late morphological changes include reduced disc height, nucleus pulposus herniation, and annular tears (4). The paraspinal muscles (PSMs) are fundamental determinants of the structural stability and function of the lumbar spine (5). There is a potential mechanism of action between defects in the vertebral endplate and decreased muscle mass of the PSMs during the development of disc degeneration. Previous studies have shown that CLPB induced myoelectric activity and muscle remodeling (e.g., muscle atrophy, fatty infiltration, and altered fiber type) (6–10). At the L4/L5 level, fatty infiltration in PSMs is more severe when damage to the adjacent spinal endplates (11). Thus the formation of IVDD is not an isolated process but a chain reaction that includes vertebral endplate changes and fatty infiltration in the PSMs (12). Given the vital role of PSMs lesions in CLBP, it is crucial to elucidate the interaction between PSMs changes and CLBP. Therefore, this article reviews the advances in the relationship and the underlying mechanisms between IVDD and PSMs fatty infiltration in patients with CLBP.

## Fatty infiltration in PSMs

The PSMs are the general term for the muscles surrounding the spine, which include the psoas, multifidus, and erector spinae. An excellent functional state of the PSMs is essential for maintaining the spine structure. A decrease in the function of PSMs can alter the original biomechanical relationships and increase the load on the disc, thus causing IVDD. Conversely, IVDD can also cause PSMs to compensate, leading to an imbalance in loads of PSMs and producing atrophy. Studies have shown that the atrophy of the PSMs is highly correlated with the degree of IVDD. Muscle degeneration is characterized by atrophy of muscle fibers, fiber bundles, and fat infiltration (13, 14). Muscle atrophy and fat replacement are thought to be the main features of PSMs remodeling in patients with CLBP, and fat infiltration may exacerbate CLBP. It is, therefore, crucial to elucidate the relationship between fatty infiltration of PSMs and IVDD.

With the development of medical imaging modalities, the metrics for assessing the atrophy of PSMs have gradually become diverse. Earlier, the degree of atrophy of PSMs was mainly determined by measuring the cross-sectional area (CSA) of PSMs using computed tomography (CT) or real-time ultrasound. In 1994, Goutallier et al. (15) proposed a semi-quantitative assessment of fatty infiltration in PSMs based on CT images, which opened new doors to exploring the mechanisms of IVDD. Using CT images, researchers found that fatty infiltration in PSMs was associated with small joint degeneration, lumbar spondylolisthesis, and narrowing of the vertebral space (16, 17). The degree of fatty infiltration in PSMs is significantly increased in patients with higher degrees of small joint degeneration (16–19). With the advent of high-resolution magnetic resonance imaging (MRI), MRI techniques have become the primary technique for assessing the atrophy of PSMs. Earlier magnetic resonance techniques used axial T2-weighted scans more often. In recent years, MRI has been able to distinguish well between muscle and adipose tissue by threshold segmentation techniques to assess the degree of atrophy of PSMs better. Ekşi et al. (20) proposed a new scoring system that included fatty infiltration in PSMs, Modic changes (MCs), and IVDD. Patients with more intense LBP had a more degenerative spine (20). However, this scoring system did not detail the role fat infiltration of PSMs played in LPB.

In 2015, Teichtahl et al. (21) used the iterative decomposition of water and fat with echo asymmetry and least square estimation-iron quantification (IDEAL-IQ) technique to quantify the fat content of PSMs and to assess the correlation between the fat content of PSMs and IVDD. They found that the fat content of PSMs was associated with reduced disc height. In addition, in 2016, the team found IVDD in all lumbar spine segments was associated with high-fat content in PSMs (22). A more recent study analyzed the correlation between fatty infiltration in different PSMs and IVDD in more detail using the Pfirmann classification (23) to assess the degree of IVDD. The study showed a strong positive correlation between Pfirmann classification and fat infiltration in the multifidus muscle (Rho=0.57, p<0.001) and a moderate positive correlation with fat infiltration in the erector spinae (Rho=0.49, p<0.001) and psoas major (Rho=0.31, p<0.001) (24).

### Multifidus

The multifidus is a general term for a group of PSMs that are shorter in cross-section but run almost the entire length of the spine and are, therefore, more susceptible to pathological changes. The multifidus is lateral to the spinous process, covering the corresponding vertebral plate, and is more closely related to the vertebral plate and spinous process than the erector spinae. Sun et al. (25) found that atrophy of the multifidus was significantly and positively correlated with IVDD at the L3/L4 disc level compared to the other PSMs. The exact mechanism of muscle degeneration is unclear. Disuse and denervation are two main mechanisms often mentioned (26). Liu et al. (27) proposed two hypothetical models by studying 264 subjects. One was that degeneration of the multifidus caused lumbar instability, which exacerbated upper lumbar disc degeneration. The other was that a herniated lumbar disc compressed the nerve roots of the corresponding segment, resulting in post-denervated multifidus atrophy. Hodges et al. (28) and Goubert et al. (29) suggested the reduction in multifidus activity due to pain as the leading cause of wasting muscle atrophy. Studies have shown that fatty infiltration in PSMs is strongly associated with high-intensity pain or disability and structural abnormalities of the lumbar spine (30). However, a study of patients with high-intensity pain and disability, which excluded the effect of physical activity level by adjusting for bias in the results, demonstrated that fatty infiltration in multifidus was an independent influence factor on the degree of disc degeneration (22). The facts about the fatty infiltration in multifidus during disc degeneration are clear. But the opposite conclusion is shown in studies targeting the muscle’s CSA. Faur et al. (26) reported that multifidus degeneration occurs mainly in the cross-section of MRI scans. However, the more common view is that muscle CSA does not correlate with IVDD (24, 31). To improve the bias caused by muscle CSA in individual body size, Urrutia et al. (32) calculated the relative CSA (RCSA) by dividing the CSA of the L3 vertebrae by the muscle CSA and showed a stronger correlation between fat signal fraction and IVDD. In addition, muscle symmetry became a perspective that was looked at. It has been suggested that a 10% or more significant asymmetry in the multifidus’ CSA be used to indicate potential spinal abnormalities (33). However, it was found that more than 10% of men with no history of LBP also had asymmetry of PSMs (34) and that asymmetry of muscle CSA was not associated with lumbar disc herniation (24). The Atrophy of PSMs is seen mainly in the inner side and deep layers of cross-sectional scans on MRI of the lumbar spine (26). Thus, measuring the CSA of PSMs and the ratio of functional CSA to CSA to assess the degree of fatty infiltration in PSMs can be biased by individual measurement differences. In addition, it may also produce inconsistent results for different lumbar spine segments. Some investigators have suggested that fatty infiltration in PSMs correlates more strongly with pathological changes in the intervertebral disc than muscle CSA (35).

Sarcopenia is defined as systemic muscle mass loss and a decline in physical performance (36), of which back muscle atrophy or fat infiltration may be a component (37). A study has shown that systemic muscle mass loss substantially impacts back muscle atrophy and fatty infiltration more than disc degeneration (37). In other words, the effect of age and gender on systemic muscle mass can further affect back muscle atrophy and fat infiltration. Another study using CT techniques to analyze trunk muscles showed that, in addition to the multifidus, fatty infiltration in the gluteus maximus and transversus abdominis muscles was also associated with IVDD (17). Still, the exact mechanisms involved need to be further explored.

Significant results have been obtained in cross-sectional studies, and experimental animal studies under univariate control are essential. By quantitative MRI, Huang et al. (38) assessed fat infiltration in PSMs of patients with discogenic LBP and rats in a novel discogenic LBP model. It was found that fatty infiltration was present in the PSMs of both LBP patients and rats and that there was a causal relationship between fatty infiltration and IVDD (38). Another study showed that dogs with higher IVDD grades had less fat infiltration in psoas and multifidus than those with lower mean IVDD degrees (39). From this, the authors speculated that the presence or severity of IVDD was not uniquely associated with fat infiltration in these muscles. A study using a porcine model showed that disc and nerve root injury might lead to a CSA reduction of the multifidus and its fatty infiltration (28). However, there was no atrophy of the multifidus following disc injury in sheep (40). This evidence challenges whether IVDD affects the characteristics of PSMs, but we are more skeptical that their relationship may not be purely causal. Özcan-Ekşi et al. (30) found that fatty infiltration in multifidus increased the probability of severe LBP fourfold. Patients with severe lumbar disc herniation were likelier to have increased fatty infiltration of the multifidus and erector spinae muscles (12). Therefore, further investigation is needed to determine whether lack of muscle strength and poor control due to fatty infiltration in the multifidus is the cause of LPB or vice versa.

### Erector spinae

Although the role of the erector spinae in the spine’s biomechanics is uncertain, its primary function is to be responsible for the flexion movement of the spine and, together with the multifidus, to maintain the stability of the lumbar spine. A cross-sectional study with Japanese subjects showed that fatty infiltration in PSMs correlated with age, and fatty infiltration of the upper lumbar erector spinae was significantly associated with LBP (41). In a separate study, the proton density fat fraction (PDFF) of multifidus and erector spinae at both L4/5 and L5/S1 levels was explored by MR techniques and analyzed the correlation with IVDD. The results showed a significant correlation between the PDFF of the PSMs and the degree of IVDD (42). This correlation also confirms that the two are mutually reinforcing processes, i.e., disc degeneration can also lead to further atrophy of the erector spinae by destabilizing the spine. However, a study on erector spinae in adolescents showed that the more intense the patient’s LBP, the less fatty infiltration in erector spinae (43). The investigators suggest that this may be an automatic compensatory mechanism for the lumbar spine during the development of LBP in adolescents and children (43).

### Psoas major

The psoas major is an essential flexor muscle of the spine and is the primary connection between the trunk and lower limbs. It contributes to the extension and general stability of the lumbar spine (44). Animal studies point to significant differences in the psoas major in comparing different degrees of disc degeneration (39). But it has also been shown that fatty infiltration in erector spinae and multifidus was significantly associated with IVDD, whereas in psoas major was not significantly associated with IVDD (12). A study explicitly analyzing degenerative changes in the psoas major and lumbar spine showed that degenerative changes in the lumbar spine, including MCs, do not alter the activity of the psoas major (45). The CSA of the psoas major at the L3/L4 and L4/L5 discs is even greater in patients with LBP compared to the healthy population. The result is inconsistent with the results of previous animal experiments. In addition, Parkkola et al. (46) found that patients with CLBP had smaller psoas major by comparison with volunteers.

In contrast, Danneels et al. (47) showed no difference in CSA of the psoas major between patients with CLBP and healthy controls. Considering that the study by Danneels et al. chose subjects who did not undergo surgery, the author speculates that the difference in results may be related to increased activity of the psoas major during treatment such as surgery. It has been suggested that gender, age, and degree of disc degeneration are independently associated with the PSMs’ fat signal fraction (FSF) (32). However, only gender and age affect the FSF of the psoas major, and the degree of disc degeneration does not alter the degree of fat infiltration in the psoas major (32). A study conducted to overcome gender bias concluded that the psoas major becomes more active in female patients with pain to stabilize the lumbar spine due to significant fat infiltration in the multifidus as a compensatory mechanism (15). Although gender is an essential factor influencing PSMs infiltration (42), this does not affect the validity of the conclusions of the above study.

## Vertebral endplate changes

MCs refer to MRI signal intensity changes in the spinal endplate and subendplate bone. The characteristics of MCs were systematically described by Modic in 1988, who concluded that MCs are caused by disc degeneration and that their pathological evolution is characterized by disc degeneration → weakening or loss of endplate protection → edema of the adjacent cancellous bone → fatty infiltration of the vertebral body → fibrosis and calcification (48). They described three different Modic types (I, II, and III). Since then, mixed Modic lesions (I/II and II/III) have also been identified, which indirectly suggests that all Modic lesions can progress from one type to another (48–50). Based on the results of previous studies, types I and II are the most common types of the lumbar spine, with the most common distribution at the L4-L5 or L5-S1 levels (48, 51). Studies have concluded that Modic type II changes are less associated with LBP (51–54). The current studies confirm that Modic type II changes are more common than type I changes (48, 49, 51, 55–57) yet remain rare in individuals without degenerative lumbar disc disease (51, 58, 59).

MCs have previously been reported to occur mainly in the lower lumbar segments (L4-L5 and L5-S1) (60, 61). In a recent study, Ekşi et al. (43) found that MCs were predominantly seen at the L1-L4 level rather than the L4-S1 level and were more common in patients with severe IVDD than in those with mild to moderate IVDD. When analyzing this association on a level-by-level basis, the authors found that severe IVDD was significantly associated with MCs at the L1-L2 and L3-L4 disc levels (43). And multifidus’ fatty infiltration in the L3-L4 and L4-L5 segments increased the risk of MCs in all lumbar parts by 8.3-fold and 9.1-fold, respectively (43). An MRI study showed that fatty infiltration in PSMs was associated with reduced disc height and MCs (31). In addition, Patients with Modic type I or I/II changes had more fatty degeneration in the lumbar PSMs (62). However, there is still considerable debate as to whether MCs precede lipoatrophy or occur after back pain.

## Molecular mechanisms of fat infiltration in PSMs

The lumbar discs and the PSMs are not only adjacent but also interconnected at the molecular and metabolic levels. IVDD is characterized by a progressive decrease in the proteoglycan and water content of the nucleus pulposus and a loss of resistance to compressive loads (63). The above mechanism is one of many, so we have sorted out the possible underlying mechanisms.

### Inflammation

Early views suggested that fatty infiltration compromised the mass of the PSMs because the adipose tissue was non-contractible (64, 65). There are currently many hypotheses for the mechanism of the relationship between fatty infiltration of the PSMs and spinal disorders, such as loss of nerve (28), chronic disuse (66), and inflammation (67). Inflammation, in particular, has been extensively studied. An experiment modeled in rats demonstrated that fatty infiltration in PSMs was closely associated with inflammation (38). Inflammation contributes to the development of pain (68) and may contribute to MCs (69). Increased reactive oxygen species (ROS) production has been reported to be associated with the differentiation of preadipocytes to adipocytes and the accumulation of adipose tissue (70). Thus, effectively mitigating cellular oxidative stress in an inflammatory environment would also block ROS-induced adipogenesis (71). In a study by James et al. (72), muscle and fat specimens were collected intraoperatively from patients with herniated discs, and gene expression was detected using a quantitative polymerase chain reaction, dividing the patients into a high-fat infiltration group and a low-fat infiltration group. The results showed high tumor necrosis factor (TNF) expression in the multifidus of subjects in the high-fat infiltration group. Another study addressing the mechanism showed that the expression levels of interleukin (IL)-1β, IL-6, IL-8, nitric oxide synthase-2 (NOS-2), and transforming growth factor (TGF)-β did not differ in severe IVDD compared to mild IVDD (24). The expression of TNF in lumbar disc tissue was significantly higher in the severe degeneration group than in the mild degeneration group (24). During the inflammatory process, TNF possesses intense pro-inflammatory activity and is closely associated with various pathological processes in IVDD (73). Some researchers have speculated that TNF may not only be a product of adipose tissue but also regulate adipogenesis (72).

Fibroblasts and preadipocytes are found in the connective tissue surrounding muscle fibers and can differentiate in response to inflammation. Adipocytes also increase following sympathetic degeneration, which is likely to occur following nerve injury. On the other hand, the dramatic increase in deoxyribonucleic acid synthesis following injury leads to the secretion of pro-inflammatory cytokines, stimulating fibroblasts, preadipocytes, and muscle precursor cells, ultimately leading to adipocyte proliferation.

Histological analysis shows that patients with LBP primarily display degeneration of the multifidus muscle, which occurs in relation to elevated inflammation, fiber size, and the ratio of fat to connective tissue (74). In addition, it was found that degenerating muscles were predominantly composed of type I fibers with less vascularity (74). Although there was no concurrent sign of atrophy at the individual fiber level, inflammatory cell density and vascular density changed in different muscle groups. In particular, inflammatory cells were significantly increased in normal skeletal muscle cells in the subgroup with 10%-50% fat infiltration, which suggests that regeneration and degeneration were out of balance in that condition (74).

### Obesity

Obesity is a pro-inflammatory state that releases cytokines such as TNF-α and IL-6. It is commonly believed that obesity is closely associated with MCs. Albert et al. suggest that it is not obesity but rather its resulting overweight that plays a vital role in the development of MCs (50). Two possible mechanisms explain this effect: 1) When the disc is stressed, matrix synthesis and proteoglycan content are reduced. The load-bearing capacity then gradually decreases. 2) IVDD or disc herniation can increase the shear forces on the vertebral endplates due to loss of the nucleus pulposus. The increased axial and torsional stresses may result in microfractures of the vertebral endplate.

LBP has been reported to be significantly associated with body mass index (BMI) (75). However, it has also been suggested that BMI is not associated with fatty infiltration in PSMs (24). A study of fatty infiltration in PSMs showed no difference in pain scores between obese and non-obese patients (76). Still, obese patients had more severe disc degeneration in the lower lumbar spine, possibly due to the increased load on the vertebral body caused by obesity (76). Subcutaneous fat tissue thickness (SFTT) is a new radiological index for assessing body fat percentage (77). Recent studies have shown that SFTT at L1-L2 level was superior to BMI in predicting severe IVDD and MCs (77, 78). A zoological study showed that a high-calorie diet did not cause disc degeneration in the vertebrae of mice (79). However, advanced glycation end products (AGEs) can lead to IVDD (80, 81). The receptor for advanced glycation end-products (RAGE) deletion inhibits systemic pro-inflammatory cytokine activity. D’Erminio et al. (79) used the RAGE knockout (RAGE-KO) model to control inflammation. They found that the effect of RAGE-KO in improving IVDD was limited and gender-related, suggesting that obesity and other sources of inflammation leading to a biomechanical overload of the lumbar spine may also have an impact (79). Another study showed that diabetes, rather than obesity, reduced the glycosaminoglycan and water content of the discs, and IVDD was associated with increased vertebral endplate thickness, reduced endplate porosity, and increased levels of AGEs (81). Due to their reduced glycosaminoglycan and water content and higher AGEs levels, the discs from diabetic rats became stiffer and had less alteration during compression (81). These findings suggest that endplate sclerosis, increased oxidative stress, and AGE/RAGE-mediated interactions may explain the high incidence of IVDD in patients with type 2 diabetes (81). Cell culture studies have shown increased palmitic acid-induced apoptosis in nucleus pulposus cells and activation of caspases 3, 7, 9, and poly (ADP-ribose) polymerase (PARP) mainly through the mitogen-activated protein kinases (MAPK) pathway, particularly the extracellular-signal-regulated kinases (ERK) pathway (82). Most obese patients have abnormally high blood lipid levels, and hypertriglyceridemia can induce IVDD independent of age and BMI (82). The results do not exclude the possibility of additional direct mechanical influences in the process of disc degeneration in humans (82).

### Conversion of hematopoietic bone marrow to fatty bone marrow

The intervertebral disc is an avascular tissue in the human body. Its nutritional supply depends on the transport of capillaries from the adjacent vertebrae. The study of Krug et al. showed that the conversion of hematopoietic bone marrow to fatty bone marrow impairs the supply of adequate nutrients to the disc cells and thus may accelerate disc degeneration (83). The MRI quantitative analysis confirmed that in the early stages of IVDD, IVDD and bone marrow fat interacted to some extent, with the severity of lumbar disc degeneration increasing with the adjacent vertebral fatty conversion (84). The relationship was particularly evident in the L4/5 lumbar segment (84). Focal fat conversion in normal hematopoietic red bone marrow may impede the transport of nutrients from the bone marrow to the end plate (85). IVDD is usually accompanied by osteoporosis, suggesting that the development of osteoporosis and IVDD may be a concomitant process (86, 87). Adipocytes and osteoblasts are derived from bone marrow mesenchymal stem cells (BMSCs). In BMSCs, there is a balance between osteogenesis and lipogenesis. If this balance is disturbed, it leads to a physiological disturbance, i.e., an increase in adipocytes in the bone marrow and decreased bone formation (79). Focal fatty degeneration of the bone marrow near the disc endplates can lead to disc degeneration by impeding the transport and metabolic exchange of nutrients essential to the disc. In addition, adipocyte growth and inflammatory edema compress the blood vessels in the confined bone cavity, further reducing blood flow (49, 88).

### Adipokines

Adipose tissue releases pro-inflammatory cytokines that have a potential role in various tissue pathologies. Cytokines such as leptin, adiponectin, and TNF produced by adipocytes have been shown to be associated with obesity and osteoarthritis (89).

Leptin regulates adipose tissue metabolism and inflammation (90) and can lead to adipocyte hypertrophy (91). Leptin and TNF are components of a positive feedback loop that promotes adipocyte hypertrophy (90). This cascade response could explain the rapid deterioration of adipose infiltration over time. Segar et al. (92) found that leptin acting alone or in concert with TNF-α, IL-1β, or IL-6 in the nucleus pulposus significantly increased nitric oxide (NO) production and promoted inflammatory cytokines and matrix metalloproteinases (MMP). These processes further initiate the degradation of disc cells and the inflammatory cascade response, thereby accelerating the degenerative process (92). Meanwhile, a study by Han et al. (93) confirmed that leptin expression was associated with the calcification of the cartilage endplates.

Adiponectin, mainly produced by lipids, is downregulated in patients with disc degeneration (94). Adiponectin may play an anti-inflammatory role in maintaining the homeostasis of the degenerating disc environment by down-regulating TNF-α production by degenerating nucleus pulposus cells (94). And adiponectin can reduce TNF-α and IL-6 significantly upregulated by IL-1β stimulation in nucleus pulposus cells and annulus fibrosus cells (95). James et al. (72) found increased expression of lipocalin and NOS-2 in epidural fat. And high leptin and low arginase 1 expressions were found in the intramuscular and subcutaneous adipose tissues (72). They speculated that disc disease is associated with a dysregulation of the local inflammatory condition (72).

Resistin is commonly involved in intra-articular angiogenesis and the inflammatory milieu (96, 97). Resistin expression is upregulated in degenerating disc tissue. In nucleus pulposus cells, it binds to Toll-like receptor 4 *via* the p38-MAPK and NF-KB signaling pathways, leading to inflammation (98), further leading to metabolic disturbances in nucleus pulposus cells, and accelerated disc degeneration processes (99).

Visfatin is secreted by visceral adipocytes and is involved in immunity, stress, and inflammation processes. In degenerated disc tissue, visfatin expression levels were progressively upregulated as degeneration progressed (100). In the nucleus pulposus cells, increased visfatin expression was associated with an upregulation of degradation-related proteins (100). In contrast, the knockdown of visfatin expression or the use of inhibitors showed a decrease in cellular autophagy and a downregulation of autophagy-related protein expression (100). Similarly, a study modeled in rats to simulate severe IVDD and performed pathway analysis indicated that inhibition of visfatin protected the nucleus pulposus from degeneration and that focusing on epidural lipids and visfatin would be a potential therapeutic target to control the inflammation associated with IVDD (101).

## Conclusion

IVDD is the leading cause of CLBP. IVDD is a chronic, multifactorial, irreversible process that severely compromises spinal stability and disc shock absorption. The PSMs are fundamental determinants of the structural stability and function of the lumbar spine. Studies have confirmed that fatty infiltration in PSMs plays a crucial role in IVDD. Inflammation, obesity, conversion of hematopoietic bone marrow to fatty bone marrow, and adipokines may be potential mechanisms for fat infiltration in PSMs. However, the quantitative methods and determination criteria for fat infiltration in PSMs and the vertebral plate need to be further studied. The biochemical and molecular mechanisms of fat infiltration in IVDD remain to be further investigated. The communication between the two at the molecular level still needs to be confirmed, especially concerning the potential signaling pathways in adipocytokines in IVDD.

## Author contributions

All authors listed have made a substantial, direct, and intellectual contribution to the work and approved it for publication.
